# Research on the mechanism of academic stress on occupational burnout in Chinese universities

**DOI:** 10.1038/s41598-024-62984-2

**Published:** 2024-05-28

**Authors:** Jifeng Cao, Tongliang Dai, Hua Dong, Jingyuan Chen, Yuejin Fan

**Affiliations:** 1 Business School, Qingdao University, Qingdao, China; 2 Business School, Qingdao University of Science and Technology, Qingdao, China

**Keywords:** Academic stress, Occupational burnout, Job satisfaction, Relative deprivation, Equity theory, Jod demand-control model, Human behaviour, Social anthropology

## Abstract

In recent years, with the unremitting advancement of higher education reform, academics have been experiencing stress associated with conducting scientific research. In this study focusing on university teachers in China, we adopted a stepwise regression method and reviewed related literature to construct a mechanism of academic stress and occupational burnout. Specifically, we tested job satisfaction and relative deprivation as mediating and moderating variables and conducted empirical research on 1239 teachers from 15 universities in eastern, central, and western China. Our findings show that: (1) academic stress has a significant positive effect on occupational burnout; (2) job satisfaction has a partial role as the intermediary agent between academic stress and occupational burnout; and (3) relative deprivation positively moderates the relationship between academic stress and job satisfaction, indicating that teachers in universities and colleges are also affected by relative deprivation and the perception of inequity. These findings have significant value in the management of higher education and academic research.

## Introduction

China has implemented the “Double First-class Initiative,” a national university improvement strategy that aims to create world-class universities and first-class disciplines. As a result, performance management system reform has been required in domestic colleges and universities to transform academic research achievements into a critical index that can be used to measure the level and ranking of universities. Consequently, there has been a substantial increase in the stress experienced by university teachers^1^. “Publish or perish,” “pre-employment and prospective employment,” “achievement-determined salary,” “post appointment,” and other similar types of policies have been widely implemented in many universities and colleges^2–4^. According to a survey conducted by Mycos University on the living conditions of university teachers in 2018, the stress derived from undertaking academic research and producing publications accounted for 70% of all the stress they experienced^5^. It has also been found that academic stress generates several negative outcomes, such as academic misconduct^6,7^, reduced job satisfaction, and ultimately disturbance of the professional attitudes of teachers.

The relationship between work stress and occupational burnout has invariably gained importance in management and psychological studies^8,9^. However, research in the context of colleges and universities is relatively rare. And the context may affect the results^10,11^. First, there are differences between the stress associated with academic research and work stress in enterprises. The stress experienced in academia is often associated with workers being required to demonstrate knowledge innovation, ultra-long endurance (on projects of up to several years), diversity (from scientific research to teaching to relationships, etc.), multiplicity (compound and superposition), and other factors. Whereas the stress experienced in enterprises is often associated with periodicity and events, for example. Second, the academic workplace is not homogenous; thus, neither is the type nor level of stress experienced. For example, discrepancies within the academic workplace exist among groups, requirements, knowledge content, and other distinct factors. Finally, the current policy adjustments that are affecting talent recruitment in universities are leading to substantial improvements in the treatment of young teachers. Hence, it is important to determine whether such changes will create a relative sense of deprivation in senior teachers and whether this sense of deprivation will reduce the teachers’ enthusiasm for work. In addition, it is also worth examining whether the proposed relative sense of deprivation is felt by teachers in general, who typically express a selfless dedication to their work.

Therefore, this study raises two questions worth exploring. First, whether there is a significant correlation between academic stress and job burnout in the new context of research pressure. In this study, stepwise regression method was used to explore the relationship and mechanism. Second, teachers as a selfless dedication group, whether relative deprivation exert moderating effect on the relationship between academic stress and job satisfaction. By constructing a main effect model of academic stress, job satisfaction, and occupational burnout, this study introduces relative deprivation as a moderating variable to measure its moderating effect on academic stress and job satisfaction. The goal was to help decode the “black box” of the interplay between academic stress and occupational burnout and clarify its mechanism, so that theoretical reference can be provided to decrease teachers’ occupational burnout practically and further activate their work vitality.

## Theoretical background and hypothesis

### Academic stress and occupational burnout

The general definition of stress is the response of an individual when he or she realizes that the demands or impacts from some external factors outweigh his or her abilities^12^. The stress of academic research in universities is defined as the teachers’ awareness that the demands of scientific research are beyond their abilities, resulting in psychological and physiological reactions^4^. Occupational burnout is a situation in relevant professional fields in which individual dehumanization, emotional exhaustion, and a sense of inefficiency exist. This conceptualization classifies occupational burnout into three categories: dehumanization (indifferent relationship), emotional exhaustion (emotional fatigue), and a sense of inefficiency (a decrease in confidence and tendency for negative self-evaluation)^13^.

The Job Demands–Resources Model emphasizes that every type of job possesses factors that affect a worker’s working conditions and physical and mental health. Job requirements such as role conflict, workload, and so on, can damage a worker’s health, and the stress associated with academic research, which is a common professional requirement for university teachers, is further enhanced by university and subject evaluations. A rapid increase in stress will, to a certain extent, result in emotional fatigue among university teachers and even damage their health^14^; emotional exhaustion is one of the important predictors of job burnout^13^. Furthermore, studies have shown that work stress is an important indicator of employee deviant behavior^15^ and that excessive stress leads to poor physical and mental well-being, which results in a series of counterproductive behaviors^16^; In addition, in the face of layers of scientific research assessment requirements, the complex process of high-quality journal submission, and homogenized scientific research competition^17^, the difficulty of college teachers' submission has gradually increased, and the phenomenon of frequent rejection has become more frequent, causing teachers' scientific research confidence to be quite hit^6^, and then produce a sense of powerlessness and burnout. Therefore, when the academic stress of university teachers reaches a certain level, it tends to result in the “high quantity but low quality” problem^4^ and in detrimental behaviors, such as psychological escape, risk taking, and giving up^1,18^. Thus, when university teachers experience academic stress, they are prone to occupational burnout.

Based on this background information, Hypothesis 1 (H1) of this study was as follows:

#### H1

Academic stress exerts a significant positive effect on occupational burnout.

### Job satisfaction as an intermediary agent

Job satisfaction, as a type of subjective gratification, refers to an individual’s attitude and subjective feelings toward the current work environment, content, relationship, salary, and other factors^19^. When a worker has high job satisfaction, they are generally satisfied with the abovementioned factors (job content, work environment, colleague relationships, pay, etc.) and can maintain a positive attitude toward work. When job satisfaction is low, it means that a worker is less satisfied with the comprehensive evaluation of the current work situation and other factors and that they are easily bored at work. Hence, job satisfaction is an emotional response to different aspects of work^20^ and a judgment based on emotional comparison. Thus, job satisfaction can have a negative impact on occupational burnout (especially the dimension of emotional exhaustion).

The implementation of the Double First-class Initiative in China has resulted in the requirements for academic research becoming more demanding. In addition, research achievements have become a crucial index in the promotion process for university teachers. Given the long-term and increasing stress associated with conducting academic research, a large number of teachers are bound to be trapped in a serious state of anxiety^4^ and even suffer from insomnia, hair loss, and other physiological conditions.

With dual negative stimulation by psychological and physiological factors, it is clear that university teachers’ job satisfaction may decline. Therefore, we concluded that academic stress may have a negative impact on job satisfaction, leading to negative psychological and physiological effects and occupational burnout. Based on this analysis, the following hypotheses were proposed:

#### H2

Academic stress exerts a negative effect on job satisfaction.

#### H3

Job satisfaction exerts a negative effect on occupational burnout.

#### H4

Job satisfaction partially mediates the relationship between academic stress and occupational burnout.

### The regulation effect of relative deprivation

Relative deprivation refers to the cognition that individuals or groups believe that they are in a relatively disadvantageous position through longitudinal or horizontal comparison with the reference, which leads to dissatisfaction or even anger^21^. It is apparent that relative deprivation is the emotional experience after comparison. With the implementation of the Double First-class Initiative, universities have begun to gradually improve the treatment of newly hired academics. Many university management teams also attach great importance to young people with doctorates and believe that excellent doctors with potential are vital to the long-term competitiveness of their universities. Hence, there is a growing divide between the treatment of new teachers and that of many veteran teachers. When new teachers are treated the same as or better than many well-experienced teachers, a sense of injustice can arise, which undoubtedly creates a strong sense of deprivation in many well-experienced teachers.

According to Adams’ equity theory^22^, when an individual compares his or her own reward with other people’s rewards, if the input–output ratio of the individual is lower than that of other people, there tends to be a perception of unfairness. In the academic situation, the intensity of the introduction of new talent and the better treatment of novice teachers compared to that of many senior teachers may create the perception of injustice and of relative deprivation^23^. The increased sense of deprivation may lead to exasperation, dissatisfaction, and other negative emotions, as well as pernicious behaviors, such as disorder flow^24^ and turnover^25,26^.

In the university context, academic stress, coupled with the sense of deprivation caused by environmental and policy changes, is bound to have a negative impact on the job satisfaction of university teachers. Through H2, we have articulated that academic stress has a significant negative effect on job satisfaction. Following on from this, when an individual’s sense of relative deprivation is high, the individual perceives unfairness toward his or her own status. As a result, H5 was proposed and tested in this study:

#### H5

**H5**

Relative deprivation exerts a positive moderating effect on the relationship between academic stress and job satisfaction.

**Figure 1:**
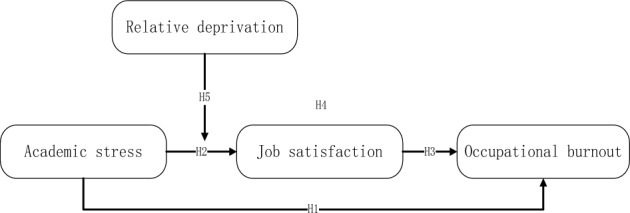
The theoretical model.

Based on the analysis of the research hypotheses above, the research model is drawn, as shown in Fig. 1.

## Materials and methods

### Sample selection and data collection

Full-time teachers engaged in scientific research and teaching for more than 5 years were the subjects in this study. The samples were selected from 15 universities in three different regions of eastern, central, and western China from September to November 2023. A total of 1400 questionnaires were distributed, and 1288 were collected, which was a collection rate of 92%, because we obtained the questionnaires through official release. Through the validity processing of the obtained questionnaires, the questionnaires with obvious errors, omissions, and a completion time of less than 100 s were deleted, leaving a total of 1239 valid questionnaires. Detailed demographic information from the participants is presented in Table 1.

**Table 1:** Overview of the participants.

Survey item	Distribution			
Gender ratio	Male		Female	
	46.96%		53.04%	
Age composition (years)	Under 29	30–39	40–49	50–61
	0	38.58%	45.44%	15.98%
Educational background	Doctoral and above	Postgraduate	Undergraduate	Vocational and under
	62.37%	29.96%	7.43%	0.24%
Teaching experience (years)	5–10	11–20	21–30	More than 31
	19.69%	44.55%	24.78%	10.98%

### Variables and measurements

The scales employed in this study are foreign and domestic scales. In the application of the foreign scale, we invited one student with a doctoral degree in business administration and two students with a master’s degree in English to conduct the research using a “literal translation and back translation” methodology. Through repeated work and continuous revision of the expression of the measurement items, they were finally applied in this study. The scales were used to assess four factors: academic stress, job satisfaction, occupational burnout, and relative deprivation.

The scale developed by Kohn and Frazer^27^ was adopted to assess academic stress. There are 14 basic items in this scale, including “I often feel pressed for time in scientific research work.”

Job satisfaction was assessed using a scale based on the Minnesota Satisfaction Questionnaire (MSQ)^28^. This scale, through analysis, can be simplified into “internal satisfaction” and “interpersonal satisfaction,” with a total of five basic items. Such as “I have a very harmonious relationship with my colleagues.

To assess occupational burnout, this study adopted the scale developed by Maslach and Jackson^13^, which contains 22 basic items, including “I feel exhausted when the workday is coming to an end,” “I feel exhausted because of my work at universities,” “Working with others directly brings me stress,” and “I feel I am overwhelmed by my work.” Based on the scale developed by Tropp and Wright^29^, the scale used to assess relative depravation has three questions, including “I think I am treated worse than others” and “I think I am treated unfairly.”

In terms of the controlled variables, the demographic variables of gender, education background, age, and teaching age were selected as controlled variables. A Likert 5-point scale was used to score each item, with “1” indicating strong disagreement and “5” indicating strong agreement.

## Data analysis and results

### Research result

The variables included in this study were found to have high internal consistency. The internal consistency coefficient of academic stress was 0.854, of relative deprivation was 0.872, of job satisfaction was 0.703, and of occupational burnout was 0.805. The internal consistency coefficients were all found to be greater than 0.7, indicating that the measurement scale used is reliable (Table 2).

**Table 2:** Descriptives and correlations.

Variable	1	2	3	4	5	6	7	8
1. Gender	NA							
2. Age	0.022	NA						
3. Education	− 0.105*	0.322**	NA					
4. Teaching Age	0.011	− 0.764**	− 0.391**	NA				
5. Academic Stress	− 0.031	0.074**	0.072*	− 0.056*	**0.854**			
6. Relative Deprivation	− 0.148**	0.045	− 0.054	0.051	0.520**	**0.872**		
7. Job Satisfaction	− 0.021	0.103**	0.073*	− 0.122**	− 0.071*	− 0.386**	**0.703**	
8. Occupational Burnout	− 0.167**	− 0.015	0.006	0.028	0.357**	0.404*	− 0.033*	**0.805**
Mean value	1.53	2.29	3.55	2.65	3.57	2.79	3.24	2.71
Standard deviation	0.50	0.76	0.64	1.11	0.60	0.92	0.58	0.39

**means *p* < 0.01; *means *p* < 0.05; the bold underlined numbers are the internal consistency coefficients of each variable; NA means “not applicable”.

To test the discriminant validity of the variables, AMOS20.0 was used to conduct confirmatory factor analysis on academic stress, relative deprivation, job satisfaction, and occupational burnout (Table 3). By comparison, it was found that the four-factor model had the best fitting effect, with *X*^*2*^*/df* (1239) = 1.715, *RMSEA* = 0.063, *CFI* = 0.902, *TLI* = 0.891, and *SRMR* = 0.053. At the same time, Harman univariate analysis was used to test the common method bias. Three basic factors were generated by exploratory factor analysis (without rotation), which explained 64.75% of the variance, among which the first factor explained 26.28% of the variance. According to the 50% judgment criterion of Harrison et al.^30^, 26.28% of the variation met the requirements, and thus common method deviation was not an issue in this study.

**Table 3:** Confirmatory factor analysis of different models.

Model	Factor	*X*^*2*^*/df*	*RMSEA*	*CFI*	*TLI*	*SRMR*
Four-factor Model	AS, JS, OB, RD	1.715	0.063	0.902	0.891	0.053
Three-factor Model	AS + JS, OB, RD	3.541	0.108	0.818	0.801	0.076
Three-factor Model	AS, JS + RD, OB	7.040	0.168	0.629	0.634	0.123
Three-factor Model	AS, JS + OB, RD	7.812	0.172	0.521	0.483	0.185
Single-factor Model	AS + JS + OB + RD	9.340	0.185	0.447	0.406	0.206

“+ ” represents the combination of two factors into one factor; AS: academic stress; JS: job satisfaction; OB: occupational burnout; RD: relative deprivation.

### Descriptive statistics

As shown in Table 2, there was a significant positive correlation between academic stress and occupational burnout (*r* = 0.357, *p* < 0.01) and a significant negative relationship between academic stress and job satisfaction (*r* = − 0.071, *p* < 0.05) and between job satisfaction and occupational burnout (*r* = − 0.033, *p* < 0.05). These findings provide preliminary support for the proposed hypothesis.

### Regression testing results

Stepwise hierarchical regression was used to test the mediating and moderating effects of the four factors and thus the hypotheses. In terms of the main effects, H1 (that academic stress exerts a significant positive effect on occupational burnout) was initially tested. Occupational burnout was set as the dependent variable, and the controlled variables included gender, teaching age, age, and education background. The independent variable of academic stress was then added to the regression equation. The regression results presented in Table 4 show that academic stress was found to have a significant positive impact on occupational burnout (M5, *ß* = 0.227, *p* < 0.01). Therefore, the data support H1.

**Table 4:** Hypothesis testing results.

	Job satisfaction				Occupational burnout		
	Model 1	Model 2	Model 3	Model 4	Model 5	Model 6	Model 7
Controlled variable							
Sex	− 0.020	− 0.023	− 0.103**	− 0.106**	− 0.130**	− 0.123**	− 0.123**
Age	0.017	0.022	0.007	0.006	0.015	0.001	0.001
Education	0.025	0.029	− 0.010	− 0.012	− 0.001	− 0.012	− 0.012*
Teaching age	− 0.050	− 0.049	− 0.044	− 0.046	0.018	0.015*	0.015*
Independent variable							
Academic stress		− 0.079**	− 0.168**	− 0.177**		0.227**	0.221**
Mediating variable							
Job Satisfaction							-0.003*
Moderating Variable							
Relative Deprivation			0.309**	0.333**			
Interaction Term							
Academic Stress x Relative Deprivation				-0.050**			
R^2^	0.016	0.023	0.187	0.195	0.029	0.155	0.155
F	5.064**	5.733**	47.326**	42.649**	9.305**	45.129**	37.583**
ΔR^2^	0.016	0.007	0.165	0.008	0.029	0.155	0.000

*means* p* < 0.05; **means *p* < 0.01.

The mediating effect of job satisfaction was then examined. We used the analytical procedure proposed by Kenny and Baron^31^ to analyze the mediating effect of job satisfaction on academic stress and occupational burnout. On the basis of the main effect analysis, when the independent variable of academic stress and the mediating variable of job satisfaction were included in the regression equation in model 7, the results showed that job satisfaction had a significant negative predictive effect on occupational burnout (M7, *ß* = − 0.003, *p* < 0.05); thus, H3 was supported by this finding. Academic stress also had a significant predictive effect on occupational burnout; however, the predictive effect was smaller (M7, *ß* = 0.221, *p* < 0.01), and the findings supported H4.

Next, we examined the moderating effect. H5 proposed that relative deprivation exerts a significant positive moderating effect on the relationship between academic stress and job satisfaction. To test this hypothesis, job satisfaction was set as the dependent variable, and the controlled variables included gender, teaching age, age, and education background. The independent variable (academic stress), moderating variable (relative deprivation), and the product of the interaction term between the moderating variable and independent variable were also added. To avoid collinearity, the independent and moderating variables were standardized before the product term was constructed. The analytical results are shown in Table 4, and they demonstrate that there was a significant negative correlation between academic stress and job satisfaction (M2, *ß* = − 0.079, *p* < 0.01), supporting H2. The interaction between relative deprivation and academic stress was found to result in a negative impact on job satisfaction (M4, *ß* = − 0.050, *p* < 0.01), indicating that the higher the sense of relative deprivation, the stronger the negative relationship between academic stress and job satisfaction, thus supporting H5.

**Figure 2:**
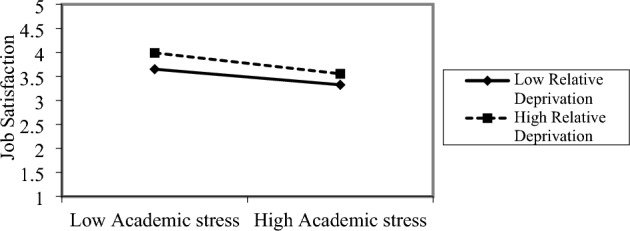
The moderating effect of relative deprivation on the relationship between academic stress and occupational burnout.

To further clarify the moderating effect of the interaction term between academic stress and relative deprivation, we took one standard deviation above and below the mean as the moderating effect of relative deprivation on academic stress and job satisfaction (Fig. 2). As shown in Fig. 2, the higher the sense of relative deprivation, the stronger the negative relationship between academic stress and job satisfaction.

## Discussion

### Research conclusions

Based on equity theory and the Job Demands–Resources Model, we explored the relationship between academic stress and occupational burnout and investigated the moderating and mediating effects of relative deprivation and job satisfaction. Our analyses were based on 1239 valid samples obtained from university teachers. The three main conclusions are outlined below.

First, academic stress has a significant positive effect on occupational burnout. The findings verify that there is a linear rather than a curved relationship between academic stress and occupational burnout, and this also demonstrates that high-intensity academic stress tends to result in occupational burnout among university teachers. The reason is that academic stress, as a form of work stress, tends to shift individuals into a state of psychological stress^32^. If an individual cannot effectively adjust to the new state of stress, it will lead to them experiencing uneasiness and anxiety^33^, and possibly long-term insomnia and other psychological, physiological, and behavioral problems^34,35^. In addition, the overload of scientific research pressure will cause the decrease of college teachers' satisfaction, but satisfaction is the key factor affecting college teachers' self-efficacy. The decrease of satisfaction means the decrease of college teachers' self-efficacy^19,36^, and self-efficacy is the key dimension of job burnout^13^. Hence, university teachers are prone to occupational burnout due to the increasing stress associated with conducting scientific research.

Second, job satisfaction partially mediates the relationship between academic stress and occupational burnout. First of all, according to Lodahl^37^ "pressure refers to the response of an individual who is aware that the external requirements exceed his own coping ability", which means that the increase of pressure will inevitably bring about discomfort. Slight discomfort may have no obvious impact, but with the continuous increase of pressure or serious overload, it can lead to anxiety and decreased satisfaction^38^. Secondly, Studies have shown that under the effect of stress, an individual’s work attitude tends to change^39^. Under excessive pressure, such attitude change is often negative. Given that academic stress (due to, for example, meeting excessive assessment indicators) is a major component of their overall stress, it is expected that college and university teachers may exhibit a decline in job satisfaction and a sense of burnout during their career.

Third, relative deprivation exerts a positive moderating effect on the relationship between academic stress and job satisfaction. This verifies that there is no direct relationship between the sense of deprivation and morality and that college and university teachers also have a relative sense of deprivation. Job satisfaction refers to individuals' subjective satisfaction with their working environment, salary, job content and relationship. However, excessive pressure will cause great damage to physical and mental health^40^, and physical and mental damage will inevitably lead to decline of satisfaction. However, at present, young teachers in Chinese universities can get a high salary just by virtue of their papers, and senior teachers will have a sense of injustice in this situation, which will lead to a high sense of relative deprivation. Therefore, under the dual effect of research pressure and relative deprivation, the negative effect of research pressure on job satisfaction will be enhanced. In other words, the higher the relative deprivation, the lower the job satisfaction when confronted with academic stress, and the higher the job satisfaction otherwise. This finding also proves that college teachers are positively affected by the sense of deprivation: when the relative deprivation of college teachers is higher, the negative effect of academic stress on job satisfaction is stronger.

### Theoretical significance

In this study, we have focused on and verified the mechanism of academic stress on occupational burnout from the perspective of the Job Demands–Resources Model. Although other scholars have discussed the relationship between job stress and occupational burnout^41–43^, we have generated a series of novel findings. First, we used a unique and distinct subject population to examine the relationship between job stress and occupational burnout: university and college teachers. This allows further verification of the scope of the specific relationship between job stress and occupational burnout for this population. It also allows, to a certain extent, the issues experienced by the groups represented by the negatively affected participants to be remedied. Second, the stress contexts addressed in this study are unique. Previous studies attached more importance to work stress in teaching, family, society, and other aspects^44^ and failed to involve academic stress. This study included specific stress situations, which are conducive to decoding the academic stress black box. Finally, we explored the relationship between academic stress and occupational burnout from the perspective of the Job Demands–Resources Model, which can effectively expand its theoretical boundaries.

With the help of equity theory, we have elaborated on the regulatory mechanism of relative deprivation on the relationship between academic stress and job satisfaction. We have also verified that college teachers are affected by deprivation. Another unique aspect of this study is that instead of focusing on the effect of common stress on job satisfaction^45^, we focused on the responses of individuals with different reactions toward relative deprivation due to academic stress. We also utilized equity theory to explain the regulatory mechanism, which is a different approach to that used in previous studies^40^, and provide a new interpretation perspective for expounding on the moderating effect of relative deprivation.

### Enlightenment for management

The lessons for management emanating from this study are as follows. First, colleges and universities should construct reasonable assessment indicators and requirements. For example, the assessment period for papers should be appropriate and realistic, and a quality-oriented research assessment system should be established (relevance to articles). Extending the cycle can effectively relieve time-related stress, reduce the academic tendency to seek quick success and instant benefits (to some extent), improve the quality of research, and partially alleviate the occupational burnout caused by academic stress. Second, given that a certain level of academic stress may always exist, universities should endeavor to eliminate other types of stress from the lives of teachers, so as to reduce their total stress, increase their job satisfaction, and reduce occupational burnout. Third, universities should endeavor to promote the contribution-oriented salary system, which is not based on seniority, to partially alleviate the sense of relative deprivation felt by senior teachers. At the same time, senior teachers should view the university through a collective lens and support common progress through cooperation and combined strength by taking advantage of faculty resources to establish or join research teams. This will help them to avoid marginalization and reduce any real or perceived relative deprivation.

### Limitations and future prospects

In this study, the research sample consisted of teachers from colleges and universities in eastern, central, and western China. Although the sample size was large, the number of colleges and universities from which survey data were obtained was relatively small, which creates certain limitations. Therefore, in future research, the number of universities and sample size should be expanded to further improve the representativeness and reliability of the research. Second, the correlation matrix shows that both gender and years of teaching relate to some of the tested variables. We will examine these relationships in a future study. Third, samples were obtained at the same time point in this study. To further explore the reliability, samples could be obtained at different time points in future studies. Finally, we applied equity theory to explain the moderating effect of relative deprivation, and other perspectives can be considered for future analysis.

## Data Availability

The raw data supporting the conclusions of this article will be made available by the authors, without undue reservation.Contact information:  daitongliang2013@126.com.
